# Rate-Dependent Effects of Biochar on Soil Fertility and Bacterial–Fungal Communities in Maize Fields of the Black Soil Region: A Three-Year Field Study

**DOI:** 10.3390/microorganisms14071487

**Published:** 2026-07-07

**Authors:** Shuangyu Cheng, Xin Ju, Kaifeng Wang, Wu Zhang, Chenglin Gu

**Affiliations:** School of Science, Jiamusi University, Jiamusi 154007, China; 18246821597@163.com (S.C.); jxtianb@163.com (X.J.); jmsdxwkf@163.com (K.W.)

**Keywords:** biochar, black soil, soil ecosystem, maize farmland, soil fertility, microbial community, bacterial and fungal diversity, sustainable soil management

## Abstract

Biochar can improve soil physicochemical properties and microbial habitats; however, its application rate-dependent effects in maize fields of the black soil region remain insufficiently understood under field conditions. A three-year field experiment was conducted in Jiamusi, Heilongjiang Province, China, from 2023 to 2025, with four biochar application rates: 0 (W0), 10 (W1), 20 (W2), and 40 t ha^−1^ (W3). Soil physicochemical properties, bacterial communities based on 16S rRNA gene sequencing, and fungal communities based on internal transcribed spacer (ITS) sequencing were analyzed to assess changes in soil fertility and microbial community composition and their relationships with environmental factors. Biochar application significantly increased soil organic matter, alkali-hydrolyzable nitrogen, available potassium, and pH. Although W3 produced the greatest nutrient enhancement, W2 exhibited a more balanced overall response across the measured soil fertility and microbial community indicators. Sequencing depth was adequate for all samples, and bacterial alpha diversity was comparatively well maintained under W2 and W3. Fungal alpha diversity exhibited pronounced interannual variation and increased under W3 in 2025. Year accounted for a greater proportion of variation in microbial community structure than did biochar treatment; however, both treatment and the year × treatment interaction also had significant effects. Among the measured soil fertility and microbial community indicators, W2 produced a comparatively balanced overall response, whereas W3 exerted stronger selective effects on microbial communities. Because crop yield, economic feasibility, labile carbon fractions, and long-term ecological outcomes were not assessed, an agronomically optimal biochar application rate cannot yet be determined.

## 1. Introduction

Black soil is a critical soil resource in China’s major grain-producing regions and is generally characterized by high organic matter content, a well-developed aggregate structure, and strong nutrient-supplying capacity [1]. These characteristics reflect the inherent fertility potential of the black soil region rather than the current condition of every cultivated field. The Northeast black soil region is a major commodity-grain production base, and maize is one of its principal crops [2,3]. However, long-term intensive cultivation, continuous monocropping, inadequate return of organic residues, imbalanced nutrient inputs, and erosion have contributed to declines in soil organic carbon (SOC), aggregate stability, nutrient-retention capacity, and biological activity in some cultivated black soils [4,5,6]. Consequently, current high maize productivity can coexist with soil degradation: fertilizer and other management inputs may sustain short-term yields, whereas progressive carbon loss and structural deterioration may increase production costs and reduce resilience to climatic variability over the longer term.

Soil microorganisms are essential components of farmland ecosystems. They participate in organic matter decomposition, nitrogen, phosphorus, and potassium transformation, rhizosphere nutrient supply, soil aggregate formation, and crop health maintenance [7,8]. Changes in bacterial and fungal community structure therefore provide biologically meaningful indicators of shifts in soil environmental conditions and of the effectiveness of sustainable farmland management practices [9,10]. An integrated assessment of soil physicochemical properties and microbial communities is consequently important for black soil conservation, fertility maintenance, and sustainable maize production [11,12].

Biochar is a carbon-rich material produced through the pyrolysis of biomass under oxygen-limited conditions [13]. It is commonly characterized by stable aromatic carbon, a porous structure, mineral ash, surface functional groups, and variable cation-exchange capacity [14,15]. These properties can influence soil pH, water retention, nutrient sorption and retention, and microbial habitat conditions [16,17]. Although most biochar carbon is relatively stable, smaller labile fractions, surface-associated organic compounds, and adsorption–desorption processes may also alter dissolved organic carbon (DOC) availability and microbial access to substrates. Together with biochar-induced changes in pH and nutrient availability, these processes may influence microbial biomass, extracellular enzyme activity, and coupled carbon, nitrogen, and phosphorus transformations [15,18,19,20,21]. The magnitude and direction of these effects depend on feedstock type, pyrolysis conditions, application rate, soil properties, climate, and field management practices [22,23,24,25].

Biochar also provides a potential pathway for recycling agricultural residues and increasing stable carbon inputs to soil under sustainable agricultural management systems [13,21]. In the Northeast black soil region, biochar may influence crop growth and soil microbial processes by regulating pH, enhancing nutrient retention, and modifying the rhizosphere microenvironment [22,23]. Therefore, long-term field experiments are needed to determine how different biochar application rates affect soil fertility and microbial communities under realistic interannual variation, rather than under short-term incubation or pot conditions [24,25,26,27,28,29].

Soil bacteria and fungi may respond differently to biochar application [30]. Bacteria are generally more sensitive to changes in soil pH, nutrient availability, labile carbon sources, and moisture conditions. In contrast, fungi, owing to their hyphal growth form and capacity to decompose complex organic matter, may be more strongly influenced by organic carbon fractions, carbon-to-nitrogen ratios, rhizosphere conditions, and soil pore structure [31,32]. Previous studies have shown that biochar can alter bacterial and fungal community composition, affect microbial diversity, shift the relative abundance of dominant taxa, and influence functional microbial groups associated with carbon and nitrogen cycling [33,34]. These responses do not necessarily indicate uniform improvement; instead, they may reflect diversity maintenance, selective enrichment of dominant taxa, or broader community restructuring [35,36].

Three knowledge gaps remain particularly relevant to black soil maize systems. First, multi-year field evidence regarding rate-dependent biochar effects remains limited because much of the available evidence is derived from pot experiments, greenhouse trials, or short-term incubations [37]. Second, the coordinated interannual responses of bacterial and fungal communities to the same biochar application rate gradient have rarely been evaluated within a single field experiment [38,39]. Third, the relationships among soil nutrient status, pH, microbial diversity, and community reassembly require further clarification through integrated multivariate analyses [40,41,42]. Although biochar may affect active carbon processes, the present study did not directly measure DOC, microbial biomass carbon, enzyme activities, SOC fractions, or soil CO_2_ fluxes. Therefore, these processes are discussed only as plausible mechanisms rather than directly demonstrated pathways.

This study focused on a maize farmland ecosystem in the Northeast black soil region, where maintaining soil fertility is closely linked to regional food security. Using a three-year field experiment, we explicitly considered interannual variation, and compared low, medium, and high biochar application rates in terms of soil fertility enhancement, microbial community stability, and community restructuring. This design provides evidence for interpreting ecological trade-offs across the tested application rate gradient without assuming that the highest rate necessarily represents a practical management recommendation.

Accordingly, a field experiment was conducted in a maize field at Sifengshan, Jiamusi City, Heilongjiang Province, China, from 2023 to 2025, with four biochar application rates: 0, 10, 20, and 40 t ha^−1^. We evaluated soil physicochemical properties, bacterial and fungal alpha diversity, community composition, beta diversity, and differential microbial taxa. Redundancy analysis (RDA) and Spearman correlation analysis were used to examine associations between soil environmental factors and microbial community shifts. We hypothesized that biochar application would improve soil fertility and increase soil pH; that bacterial and fungal communities would respond differently to increasing application rates; and that medium-to-high application rates would induce stronger community reassembly, with the medium rate producing a comparatively balanced microbial response and the high rate exerting stronger selective pressure. Because crop yield, economic feasibility, and direct active carbon mechanisms were not assessed, this study does not define an agronomically optimal biochar application rate.

## 2. Materials and Methods

### 2.1. Study Site

The field experiment was conducted from 2023 to 2025 in a maize field at Sifeng Mountain, Jiamusi City, Heilongjiang Province, China. The experimental site was located at 45°45′35″ N and 130°22′59″ E, and the soil was classified as black soil. During the preceding decade, the region had a mean annual precipitation of 688 mm and a reported mean annual temperature of 3 °C. The site is located within a typical maize-producing area of the Northeast black soil region. The plow layer soil texture was suitable for maize cultivation, the cultivation histories of the plots were broadly comparable, and field management followed local conventional maize production practices.

Because microbial communities in open-field agroecosystems can be sensitive to interannual climatic variation, year was included as an explanatory factor in all relevant statistical analyses. This approach enabled the effects of biochar application to be assessed while accounting for interannual variation, rather than treating them as isolated single-factor responses.

Before treatment application, soil samples were collected from the 0–20 cm plow layer to determine baseline physicochemical properties. Initial soil properties were as follows: organic matter, 18.44 g kg^−1^; total nitrogen, 0.27 g kg^−1^; alkali-hydrolyzable nitrogen, 164.88 mg kg^−1^; total phosphorus, 0.83 g kg^−1^; available phosphorus, 33.11 mg kg^−1^; total potassium, 21.47 g kg^−1^; available potassium, 177.88 mg kg^−1^; and pH, 7.31. Using the conventional conversion factor of 1.724, initial SOC was estimated at 10.70 g kg^−1^ from the measured organic matter content. This value is provided solely to clarify the baseline carbon status and should not be regarded as a direct SOC measurement. These baseline characteristics provided an appropriate basis for evaluating rate-dependent responses to biochar application in black soil maize farmland.

### 2.2. Basic Properties of the Tested Soil and Biochar

Maize cultivar ‘Mengfa 9009’ was used in the field experiment. Stanley compound fertilizer was applied at an N–P_2_O_5_–K_2_O nutrient ratio of 25–12–13. The biochar was supplied by Henan Lize Environmental Protection Technology Co., Ltd (Shangqiu, China), and was produced from maize straw by oxygen-limited pyrolysis at 500 °C for 2 h. The dry-mass biochar yield was 30.2%, calculated as the dry mass of recovered biochar divided by the dry mass of input maize straw × 100. Before field application, the biochar was crushed and passed through a 2 mm sieve to facilitate uniform incorporation into the 0–20 cm plow layer. The applied biochar batch contained 447.07 g kg^−1^ organic matter, 258.7 g kg^−1^ total carbon, 6.01 g kg^−1^ total nitrogen, 5.89 g kg^−1^ total phosphorus, and 27.61 g kg^−1^ total potassium. Its pH was 8.89, ash content was 18.60%, C/N ratio was 43.10, cation-exchange capacity (CEC) was 38.1 cmolc kg^−1^, and specific surface area was 86.35 m^2^ g^−1^. The basic properties of the biochar used in the experiment are presented in Table 1.

The measured total carbon content, CEC, alkaline pH, potassium content, and specific surface area of the applied biochar provide a physicochemical basis for interpreting its potential effects on soil carbon input, nutrient retention, pH regulation, and microbial habitat conditions. However, these material properties do not demonstrate that soil CEC or active carbon fractions increased after field application, because these soil responses were not directly measured in the present study.

### 2.3. Experimental Design and Field Management

A randomized complete block design was used to minimize the influence of spatial heterogeneity across the field. The experiment included four biochar application rates, with three independent field replicates per treatment, resulting in a total of 12 experimental plots. Each plot measured 9 m^2^ (3 m × 3 m), and plots within each block were managed under comparable field conditions. The treatments were 0 t ha^−1^ (W0, control), 10 t ha^−1^ (W1, low rate), 20 t ha^−1^ (W2, medium rate), and 40 t ha^−1^ (W3, high rate). In each year, one composite soil sample was collected from each replicate plot; thus, three independent composite samples were obtained per treatment for soil physicochemical analyses and microbial sequencing.

The highest biochar rate (40 t ha^−1^) was intentionally included as an upper-bound intensification scenario to characterize the response range of soil fertility and microbial communities. It was not intended to represent a practical one-season on-farm residue-return rate. Based on the recorded dry-mass biochar yield of 30.2%, W3 was equivalent to the biochar produced from approximately 132.5 t dry maize straw ha^−1^. This mass-equivalent calculation illustrates why W3 should be interpreted as a boundary-response treatment for evaluating ecological trade-offs and potential nonlinear responses. W2 was included as an intermediate rate to determine whether a less intensive amendment could maintain soil and microbial benefits while reducing biochar input requirements and the potential for stronger community restructuring.

Before maize sowing in 2023, biochar was applied once at the designated rates to the soil surface of each plot and incorporated into the 0–20 cm plow layer by tillage. Maize was sown in early May each year; specifically, on 5 May 2023, 6 May 2024, and 5 May 2025. The crop reached maturity between late September and early October, and maturity-stage soil sampling and harvest were conducted on 28 September 2023, 29 September 2024, and 28 September 2025. Maize was planted at a row spacing of 65 cm and a plant spacing of 25 cm, corresponding to a theoretical density of approximately 61,500 plants ha^−1^.

Compound fertilizer was applied once before sowing as basal fertilizer at 750 kg ha^−1^, equivalent to 187.5 kg N ha^−1^, 90.0 kg P_2_O_5_ ha^−1^, and 97.5 kg K_2_O ha^−1^. No topdressing was applied during the maize growing season. All treatments received the same conventional local field management, except for differences in biochar application rate. This three-year field experiment was designed to assess the effects of biochar application rate on soil physicochemical properties, bacterial and fungal community structure, and their relationships with soil environmental factors in maize farmland.

### 2.4. Soil Sampling and Processing

Soil samples were collected at maize maturity in 2023, 2024, and 2025. In each replicate plot, soil was collected from the 0–20 cm plow layer at five points along a diagonal transect to reduce microsite variability. The five subsamples from each plot were thoroughly homogenized to produce one composite sample, which was treated as an independent biological replicate. Thus, each treatment was represented by three independent biological replicates per year. Across the three-year experiment, 36 composite soil samples were collected (4 treatments × 3 replicates × 3 years) and subsequently allocated to soil physicochemical and microbial community analyses. Surface residues, visible roots, stones, and other debris were removed during sampling.

Each composite sample was divided into two subsamples. One subsample was air-dried, ground, and sieved for soil physicochemical analysis. The other subsample was transferred to sterile centrifuge tubes, transported to the laboratory on dry ice, and stored at −80 °C until high-throughput sequencing of soil bacterial and fungal communities. Microbial sequencing was conducted by Shanghai Personal Biotechnology Co., Ltd. (Shanghai, China). A continuous cold chain was maintained during transport and storage to minimize microbial DNA degradation and potential shifts in community composition.

### 2.5. Determination of Soil Physicochemical Properties

Soil physicochemical properties were determined using standard agrochemical procedures after the samples had been air-dried, ground, and passed through a 2 mm sieve. Soil pH was measured potentiometrically in a soil-to-deionized-water suspension (1:2.5, *w*/*v*) using a calibrated pH meter (PHS-3E, Shanghai INESA Scientific Instrument Co., Ltd., Shanghai, China). Before measurement, the suspension was thoroughly stirred and allowed to equilibrate, and the pH meter was calibrated with standard buffer solutions.

Soil organic matter was determined using the potassium dichromate oxidation method. Briefly, air-dried soil was oxidized with potassium dichromate under acidic heating conditions, and the residual dichromate was quantified by titration. Alkali-hydrolyzable nitrogen was determined using the alkaline hydrolysis diffusion method, in which released ammonia was absorbed and quantified by acid titration. Available phosphorus was extracted with 0.5 mol L^−1^ NaHCO_3_ and determined using the molybdenum–antimony colorimetric method with a UV–visible spectrophotometer (TU-1901, Beijing Purkinje General Instrument Co., Ltd., Beijing, China). Available potassium was extracted with neutral ammonium acetate and determined using a flame photometer (FP640, Shanghai Yidian Analytical Instrument Co., Ltd., Shanghai, China).

Total nitrogen was determined using the Kjeldahl method, including sulfuric acid digestion, distillation, and titration. Total phosphorus was determined after acid digestion using the molybdenum–antimony colorimetric method with a UV–visible spectrophotometer, whereas total potassium was determined after acid digestion by flame photometry. Quality-control procedures included reagent blanks, replicate measurements, and instrument calibration before sample analysis. These indicators were used to characterize soil fertility status and its interannual variation in black soil maize farmland under different biochar application rates.

Because biochar contains a substantial proportion of stable carbon, residual biochar particles may have contributed to the measured soil organic matter content. Accordingly, increases in soil organic matter were interpreted as reflecting both exogenous biochar-derived carbon input and changes in the soil organic matter pool, rather than solely an increase in native soil organic matter.

### 2.6. Soil DNA Extraction, PCR Amplification, and High-Throughput Sequencing

For each composite soil sample, total genomic DNA was extracted from approximately 0.25 g of soil stored at −80 °C using the DNeasy PowerSoil Pro Kit (QIAGEN, Hilden, Germany), according to the manufacturer’s instructions. DNA integrity was assessed by 1% agarose gel electrophoresis, whereas DNA concentration and purity were determined using a NanoDrop 2000 spectrophotometer (Thermo Fisher Scientific, Waltham, MA, USA). Only DNA samples with adequate purity and intact electrophoretic bands were used for subsequent PCR amplification and sequencing library preparation.

For bacterial community analysis, the V3–V4 hypervariable region of the 16S rRNA gene was amplified using primers 338F (5′-ACTCCTACGGGAGGCAGCA-3′) and 806R (5′-GGACTACHVGGGTWTCTAAT-3′). For fungal community analysis, the ITS1 region was amplified using primers ITS1F (5′-CTTGGTCATTTAGAGGAAGTAA-3′) and ITS2R (5′-GCTGCGTTCTTCATCGATGC-3′).

PCR amplification was performed in 25 μL reaction mixtures containing 12.5 μL of 2× Phusion High-Fidelity PCR Master Mix (New England Biolabs, Ipswich, MA, USA), 0.5 μL each of forward and reverse primers (10 μmol L^−1^), 10 ng of template DNA, and sterile ddH_2_O to a final volume of 25 μL. Each sample was amplified in three technical replicates, and the replicate amplicons were pooled after PCR to reduce stochastic amplification bias.

The PCR program for bacterial 16S rRNA gene amplification consisted of an initial denaturation at 98 °C for 30 s; 30 cycles of denaturation at 98 °C for 10 s, annealing at 54 °C for 30 s, and extension at 72 °C for 45 s; followed by a final extension at 72 °C for 10 min. The fungal ITS amplification program consisted of an initial denaturation at 98 °C for 30 s; 30 cycles of denaturation at 98 °C for 10 s, annealing at 52 °C for 30 s, and extension at 72 °C for 45 s; followed by a final extension at 72 °C for 10 min. PCR products were verified by 2% agarose gel electrophoresis, and the target amplicons were purified and quantified.

Qualified amplicons were pooled at equimolar concentrations for sequencing library preparation. Paired-end sequencing was performed on the Illumina MiSeq PE300 platform (Illumina, San Diego, CA, USA). The resulting raw reads were subjected to subsequent bioinformatic analysis.

### 2.7. Bioinformatic Analysis

Raw sequencing reads were subjected to initial quality control to remove adapter sequences, low-quality reads, and reads containing ambiguous bases. Bacterial and fungal reads were then processed separately using QIIME 2 (version 2023.2). The DADA2 plugin was used for read denoising, paired-end read merging, chimera removal, and generation of amplicon sequence variant (ASV) feature tables. ASVs served as the fundamental analytical units for microbial diversity and community composition analyses.

Before diversity and community composition analyses, each ASV feature table was rarefied to the minimum sequencing depth within the corresponding dataset to minimize bias arising from unequal sequencing effort among samples. This normalization enabled comparisons of alpha diversity, beta diversity, and relative taxonomic composition at comparable sequencing depths.

Representative bacterial ASV sequences were taxonomically classified using the SILVA 138.1 database, whereas representative fungal ASV sequences were annotated using the UNITE 8.2 database. Taxonomic assignments were generated with the q2-feature-classifier plugin in QIIME 2 using a confidence threshold of 0.70. Because the 16S rRNA gene V3–V4 region and fungal ITS1 amplicons generally provide reliable taxonomic resolution from the phylum to genus levels, but do not consistently support confident species-level identification for all taxa, ecological interpretation focused primarily on phylum- and genus-level patterns. Species-level assignments were not used for ecological interpretation unless annotation confidence was sufficiently high. To minimize overinterpretation, ecological inferences focused on dominant and differentially enriched genera supported by the SILVA and UNITE annotations. More reliable species- or strain-level identification would require longer marker regions, full-length amplicon sequencing, metagenomic sequencing, or targeted isolation and verification.

Relative abundances of major bacterial and fungal taxa were calculated at the phylum and genus levels. Phylum-level community composition was visualized using stacked bar plots, whereas dominant genera were visualized using heatmaps.

Bacterial and fungal alpha diversity was evaluated using the Chao1, observed ASVs, Shannon, and Simpson indices. Community dissimilarity among samples was calculated using Bray–Curtis distances, and principal coordinate analysis (PCoA) was used to visualize differences in microbial community structure among treatments and years. Permutational multivariate analysis of variance (PERMANOVA) was conducted with 999 permutations based on Bray–Curtis distances to assess the effects of year, biochar application rate, and their interaction on microbial community structure.

LEfSe (version 1.1.2) was used to identify differentially enriched microbial taxa among biochar treatments. The linear discriminant analysis (LDA) score threshold was set to 3.0, with statistical significance defined as *p* < 0.05. LEfSe results were used to identify taxa enriched under specific treatments, and heatmaps of key differential genera were used to visualize relative abundance patterns across years and treatments.

### 2.8. Analysis of Relationships Between Microbial Communities and Environmental Factors

Redundancy analysis (RDA) was performed to examine relationships between soil physicochemical variables and bacterial and fungal community structure. Before RDA, genus-level relative-abundance matrices were Hellinger transformed to reduce the disproportionate influence of highly abundant taxa. To minimize multicollinearity among environmental variables, variance inflation factor (VIF) analysis was used to guide variable selection. In the initial model, the VIF values for organic matter (OM), available potassium (AK), and pH were 55.20, 29.68, and 18.06, respectively. All values exceeded the commonly used threshold of 10, indicating substantial multicollinearity. After removal of OM, which had the highest VIF value, six environmental variables were retained for subsequent RDA: AK, total phosphorus (TP), total potassium (TK), pH, available phosphorus (AP), and alkali-hydrolyzable nitrogen (AN). Their final VIF values were 5.69, 4.57, 3.41, 2.82, 2.77, and 2.55, respectively; all were ≤10.

The significance of the RDA model was assessed using 999 permutations. Spearman correlation analysis was conducted to examine relationships between dominant genera and soil physicochemical variables, and significant correlations were visualized using correlation heatmaps. To control the false-positive risk associated with multiple comparisons, *p* values were adjusted using the Benjamini–Hochberg false discovery rate (FDR) procedure. Correlations with FDR-adjusted *p* < 0.05 were considered statistically significant. Associations with raw *p* < 0.05 but FDR-adjusted *p* ≥ 0.05 were considered nominally significant before multiple-testing correction, but were not interpreted as statistically significant after FDR adjustment.

### 2.9. Statistical Analysis

Soil physicochemical properties and microbial alpha diversity indices were expressed as mean ± standard deviation (SD) or mean ± standard error (SE), as specified in the corresponding tables and figures. Linear mixed models (LMMs) were used to evaluate the effects of year, biochar application rate, and their interaction on soil physicochemical properties. Year, biochar application rate, and their interaction were specified as fixed effects, whereas block and plot identity nested within block were included as random effects to account for the randomized complete block design and repeated annual measurements from the same plots. For complementary comparison with a conventional factorial approach, two-way analysis of variance (ANOVA) was also performed using the model: Value ~ Year × Treatment. Tukey’s honestly significant difference (HSD) test was used for post hoc pairwise comparisons when significant effects were detected, with statistical significance defined as *p* < 0.05. Because the same field experiment was monitored across years, results were interpreted primarily in terms of temporal trends and treatment-associated differences rather than as evidence of simple dose–response causality.

Microbial beta diversity was quantified using Bray–Curtis dissimilarities and visualized by principal coordinate analysis (PCoA). Permutational multivariate analysis of variance (PERMANOVA) was used to assess the effects of year, biochar application rate, and their interaction on bacterial and fungal community structure. General statistical analyses of soil physicochemical properties were conducted using SPSS Statistics 26, R, and Origin 2021. Redundancy analysis was conducted using Canoco 5 and R packages, as appropriate. High-throughput sequencing data were processed using the GeneCloud platform and the QIIME 2-based bioinformatic workflows described above.

## 3. Results and Analysis

### 3.1. Effects of Biochar Application on Soil Physicochemical Properties in Black Soil Maize Farmland

**Figure 1:**
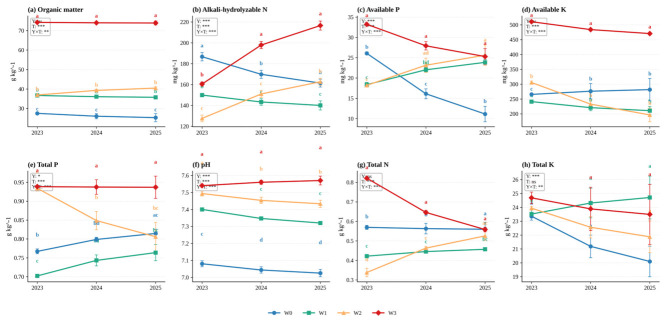
Interannual variation in soil physicochemical properties under different biochar treatments. Note: Different lowercase letters indicate significant differences among treatments within the same year (*p* < 0.05).

From 2023 to 2025, soil physicochemical properties in black soil maize farmland differed among biochar application rates and exhibited substantial interannual variation (Figure 1). Linear mixed-model results (Table 2) showed that biochar application rate had highly significant effects on organic matter, alkali-hydrolyzable nitrogen, available phosphorus, available potassium, total phosphorus, pH, and total nitrogen (*p* < 0.001), whereas its effect on total potassium was not significant (*p* = 0.734). Year had highly significant effects on alkali-hydrolyzable nitrogen, available phosphorus, pH, and total potassium (*p* < 0.001), as well as a significant effect on total phosphorus (*p* = 0.022). In contrast, year had no significant effects on organic matter, available potassium, or total nitrogen. The year × biochar application rate interaction was significant or highly significant for all measured soil properties. In particular, the interactions for alkali-hydrolyzable nitrogen, available phosphorus, available potassium, total phosphorus, pH, and total nitrogen were highly significant (*p* < 0.001). These results indicate that the effects of biochar application on soil physicochemical properties were dependent on application rate and varied across years during the three-year field experiment.

The two-way ANOVA results were broadly consistent with the linear mixed-model results, showing significant effects of biochar application rate on most measured soil physicochemical properties and significant year × biochar application rate interactions for several nutrient-related variables. These findings further support the conclusion that the effects of biochar application on soil fertility were dependent on application rate, and varied across years. Detailed PERMANOVA results for bacterial and fungal community structures are presented in Table 3.

For soil organic matter, W3 consistently exhibited the highest measured values across the three-year experiment, reaching 74.23, 74.02, and 73.91 g kg^−1^ in 2023, 2024, and 2025, respectively. These values were substantially higher than the corresponding W0 values of 27.50, 26.04, and 25.30 g kg^−1^. Soil organic matter contents under W1 and W2 were also higher than those under W0. Under W2, soil organic matter increased overall from 36.94 g kg^−1^ in 2023 to 40.46 g kg^−1^ in 2025. Notably, the large increase in measured soil organic matter under W3 may reflect both direct inputs of stable biochar-derived carbon and changes in the soil organic matter pool. Therefore, this result should be interpreted as an increase in total measured soil organic matter after biochar application, rather than as evidence solely of newly formed native soil organic matter.

Alkali-hydrolyzable nitrogen differed markedly among biochar application rates. W0 and W1 declined overall during the experiment, with W0 decreasing from 186.69 to 161.40 mg kg^−1^ and W1 from 149.91 to 140.00 mg kg^−1^. In contrast, W2 and W3 increased overall, with W2 rising from 127.57 to 162.68 mg kg^−1^ and W3 from 160.51 to 216.55 mg kg^−1^. These temporal patterns were consistent with treatment-associated increases in alkali-hydrolyzable nitrogen under the medium and high biochar application rates, particularly under W3.

Available phosphorus and available potassium also showed distinct responses to biochar application. Available phosphorus in W0 decreased from 26.08 to 11.13 mg kg^−1^, whereas concentrations under W1 and W2 increased from 18.37 and 18.20 mg kg^−1^ to 23.85 and 25.63 mg kg^−1^, respectively. These patterns indicate that the W1 and W2 treatments were associated with maintained or increased available phosphorus relative to W0. Although available phosphorus under W3 decreased from 33.22 to 25.29 mg kg^−1^, it remained higher than that under W0 throughout the experiment. For available potassium, W3 consistently exhibited substantially higher values than the other treatments, reaching 510.47, 483.88, and 470.59 mg kg^−1^ in 2023, 2024, and 2025, respectively. This pattern may partly reflect the relatively high potassium content of the maize-straw-derived biochar.

Soil pH increased across the biochar application rate gradient. In W0, pH decreased slightly from 7.08 to 7.02 over the three-year period, whereas pH in W3 increased from 7.54 to 7.57, and remained highest among the treatments. Soil pH under W1 and W2 remained within the ranges of 7.32–7.40 and 7.43–7.49, respectively. These results indicate that the alkaline biochar application was associated with higher pH in the 0–20 cm plow layer.

Overall, biochar application was associated with higher measured soil organic matter, treatment-specific changes in alkali-hydrolyzable nitrogen, available phosphorus, and available potassium, and elevated soil pH. These changes in soil environmental conditions may provide an important context for the observed shifts in microbial community structure.

### 3.2. Sequencing Data Quality of Soil Microorganisms

**Figure 2:**
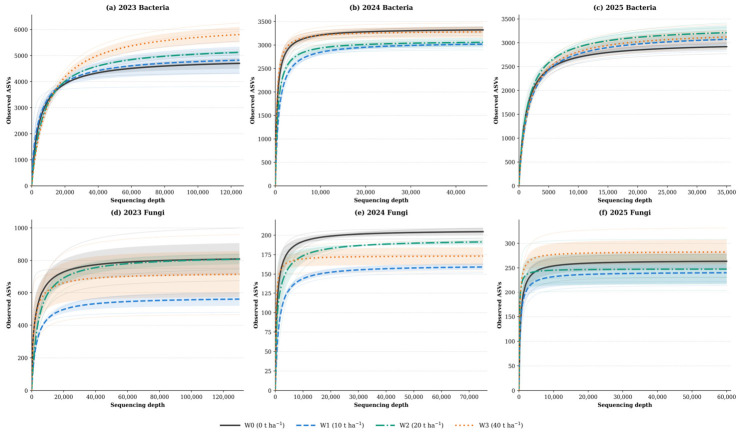
Rarefaction curves of bacterial and fungal communities from 2023 to 2025. Note: Solid curves show treatment means; shaded bands show mean ± SE; thin faint curves show individual biological replicates (n = 3).

Sequencing data from bacterial and fungal communities across biochar application rates from 2023 to 2025 showed adequate coverage (Table 4; Figure 2). Good’s coverage values for bacterial samples were ≥0.98, whereas those for fungal samples were approximately 0.999, indicating that sequencing effort was sufficient to represent the dominant bacterial and fungal taxa in the sampled soils.

Table 4 summarizes bacterial and fungal sequencing characteristics separately for 2023, 2024, and 2025. In 2023, bacterial effective sequence counts ranged from 90,976 to 115,063, and mean ASV richness values ranged from 4654.4 to 5773.9. In 2024, bacterial effective sequence counts ranged from 39,211 to 43,738, with mean ASV richness values ranging from 3016.3 to 3319.1. Following rarefaction in 2025, bacterial sequencing depth was standardized at 34,134 reads per sample, and mean ASV richness values ranged from 2917.3 to 3209.7. Fungal sequencing data also exhibited high coverage, although fungal ASV richness varied among years and biochar application rates. Presenting the sequencing characteristics separately by year allows sequencing output and microbial richness to be interpreted in the context of interannual variation.

Rarefaction curves showed that ASV richness increased with sequencing depth and approached a plateau as the number of reads increased, indicating that additional sequencing would be expected to yield relatively few additional ASVs. These results suggest that sequencing depth was sufficient to capture most detectable bacterial and fungal diversity in the soil samples. Therefore, the sequencing data were adequate for subsequent analyses of microbial community structure in black soil maize farmland across different biochar application rates.

### 3.3. Effects of Biochar Application on Soil Bacterial Alpha Diversity

**Figure 3:**
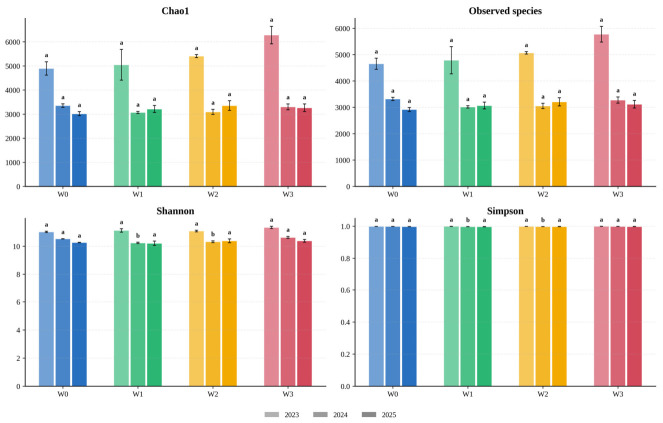
Alpha diversity indices of the bacterial community from 2023 to 2025. Note: Different lowercase letters within the same year indicate significant differences among treatments (*p* < 0.05).

Bacterial alpha diversity patterns differed among biochar application rates and varied across years (Figure 3). In 2023, bacterial alpha diversity indices were generally high, and Chao1, observed ASVs, Shannon, and Simpson indices were comparatively high under W3. This pattern was consistent with relatively greater bacterial richness and diversity under the high biochar application rate during the first year of the experiment. In 2024, bacterial alpha diversity was generally lower than in 2023, and differences among biochar application rates were less pronounced. Nevertheless, W3 retained comparatively high Shannon and Simpson indices, indicating relatively high bacterial diversity and evenness under the high application rate.

In 2025, bacterial alpha diversity again differed among biochar application rates. Chao1, observed ASVs, and Shannon indices were higher under W2, whereas the Simpson index was higher under W3. These results suggest that, after three years of field application, the medium and high biochar rates were associated with comparatively high bacterial alpha diversity, although the responses differed among diversity indices. Overall, bacterial alpha diversity did not exhibit a linear response to biochar application rate; instead, it varied according to both application rate and year. In the context of the observed changes in soil physicochemical properties, the medium and high biochar rates may have contributed to soil conditions associated with shifts in bacterial diversity. However, the present results do not directly demonstrate the specific mechanisms underlying these associations.

### 3.4. Effects of Biochar Application on Soil Fungal Alpha Diversity

**Figure 4:**
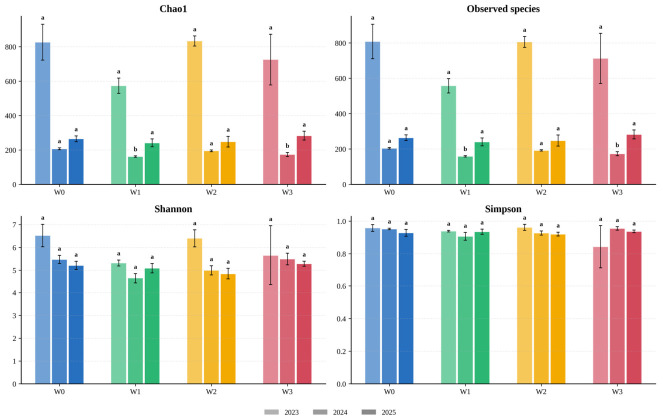
Alpha diversity indices of the fungal community from 2023 to 2025. Note: Different lowercase letters within the same year indicate significant differences among treatments (*p* < 0.05).

Fungal alpha diversity patterns showed greater interannual variation than bacterial alpha diversity, and differed among biochar application rates (Figure 4). In 2023, fungal richness was comparatively high, and Chao1 and observed ASV indices were higher under W0 and W2. This pattern was consistent with relatively high fungal richness under the medium biochar application rate during the first year of the experiment. In 2024, fungal alpha diversity was lower across all treatments than in 2023, indicating substantial interannual variation in fungal community diversity. In 2025, fungal richness partially recovered relative to 2024. In particular, W3 exhibited higher Chao1, observed ASV, and Shannon indices, indicating comparatively high fungal richness and diversity under the high biochar application rate in the third year.

Overall, fungal alpha diversity exhibited pronounced interannual variation. The response pattern differed across years: W2 was associated with relatively high fungal richness in the first year, whereas W3 was associated with comparatively high fungal richness and diversity in 2025. These patterns may be related to temporal changes in soil conditions following biochar application. However, because soil carbon fractions, pore characteristics, and rhizosphere processes were not directly measured, the specific mechanisms underlying the observed fungal community responses cannot be determined from the present data.

### 3.5. Effects of Biochar Application on Soil Bacterial Community Composition

**Figure 5:**
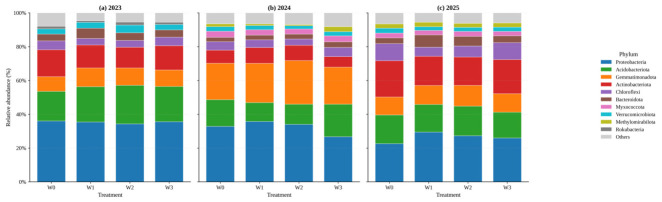
Bacterial community composition at the phylum level.

From 2023 to 2025, bacterial communities in black soil maize farmland were dominated by Proteobacteria, Acidobacteriota, Actinobacteriota, Gemmatimonadota, Chloroflexi, and Bacteroidota (Figure 5). Proteobacteria was the most abundant phylum in all three years, although its mean relative abundance declined over time. In contrast, the mean relative abundances of Actinobacteriota and Chloroflexi were higher in 2025, whereas Gemmatimonadota showed a pronounced increase in 2024. These results indicate that the relative abundances of dominant bacterial phyla varied across years and among biochar application rates. However, because no phylum-level evenness or compositional balance metric was evaluated, the observed patterns should not be interpreted as definitive evidence of a shift toward a more balanced bacterial community.

**Figure 6:**
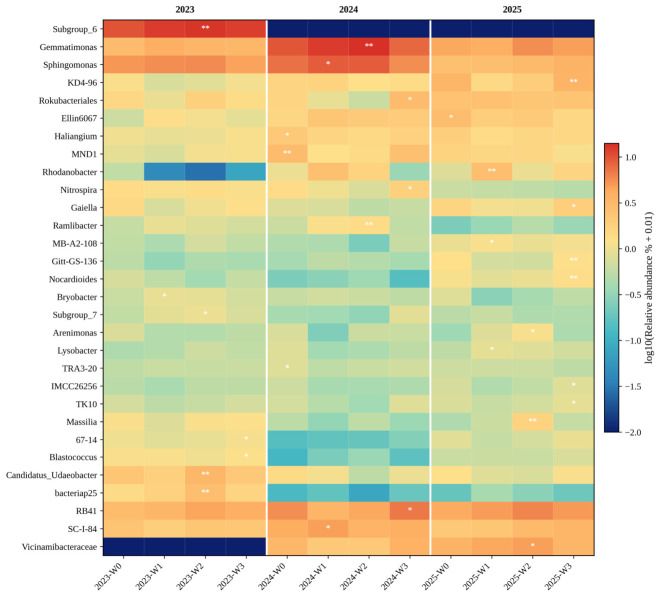
Heatmap of dominant bacterial genera. Note: Asterisks mark the treatment-year combination with the highest relative abundance for each genus.

Genus-level heatmaps further indicated that Gemmatimonas, Sphingomonas, RB41, SC-I-84, KD4-96, Rokubacteriales, MND1, Haliangium, Rhodanobacter, Nitrospira, and other dominant bacterial taxa varied across biochar application rates and years (Figure 6). Gemmatimonas and Sphingomonas exhibited comparatively high relative abundances under W1 and W2 in 2024, whereas RB41 was relatively abundant under W2 in 2025. KD4-96 and Nitrospira showed comparatively high relative abundances under W3 in 2025. These patterns indicate that medium and high biochar application rates were associated with shifts in the relative abundances of several dominant bacterial taxa. The ecological functions of these taxa cannot be directly inferred from 16S rRNA gene amplicon data alone. Functional interpretations based on taxonomic annotations and the published literature should therefore be verified using functional gene analysis, metagenomic sequencing, enzyme activity measurements, or targeted isolation and characterization.

### 3.6. Effects of Biochar Application on Soil Fungal Community Composition

**Figure 7:**
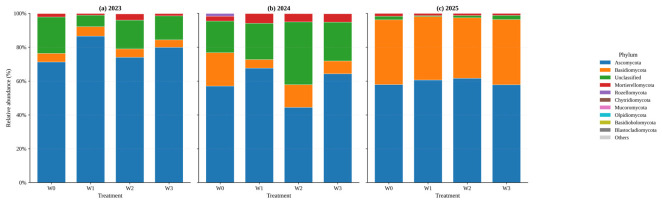
Fungal community composition at the phylum level.

Soil fungal communities were dominated by Ascomycota, Basidiomycota, and Mortierellomycota (Figure 7). Ascomycota was the most abundant phylum in all three years, although its mean relative abundance was lower in 2024 and 2025 than in 2023. In contrast, the mean relative abundance of Basidiomycota was higher in 2025. These results indicate that the relative abundances of the dominant fungal phyla varied across years and among biochar application rates. However, because no phylum-level evenness or compositional balance metric was evaluated, these patterns should not be interpreted as definitive evidence of a shift toward a more balanced fungal community.

**Figure 8:**
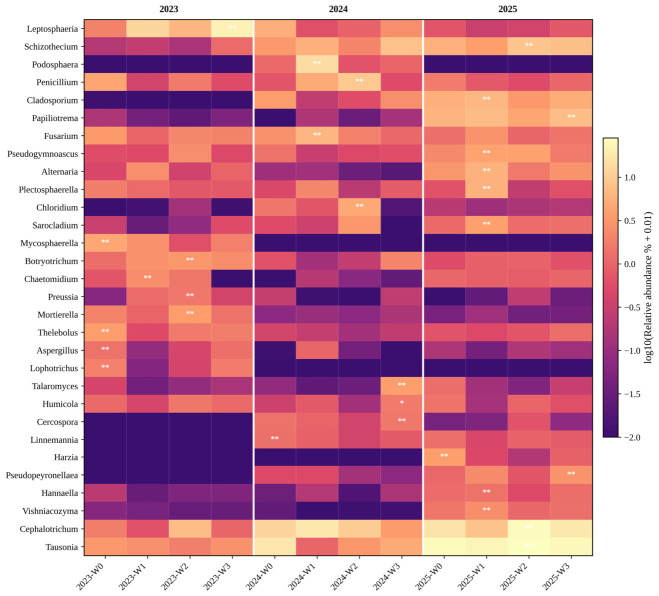
Heatmap of dominant fungal genera. Note: Asterisks mark the treatment-year combination with the highest relative abundance for each genus.

Genus-level heatmaps indicated that several dominant fungal taxa, including Leptosphaeria, Tausonia, Cephalotrichum, Podosphaera, Penicillium, Mortierella, Humicola, Chaetomidium, Mycothermus, Thermomyces, and Solicoccozyma, varied across years and biochar application rates (Figure 8). In 2023, Leptosphaeria exhibited a comparatively high relative abundance under W3. In 2024 and 2025, Tausonia and Cephalotrichum showed comparatively higher relative abundances in several treatments, consistent with substantial interannual variation in fungal community composition. Mortierella and several taxa assigned to Basidiomycota also showed relatively high abundances in biochar-amended treatments. However, trophic roles and decomposition functions cannot be directly inferred from taxonomic annotations alone. Therefore, potential relationships between these taxa, organic carbon transformation, and fungal niche differentiation require verification using functional gene analysis, metagenomic sequencing, enzyme activity measurements, or targeted isolation and characterization. Species-level and functional-guild interpretations should consequently be made cautiously.

Fungal communities exhibited pronounced compositional variation at the phylum and genus levels across years. The lower relative abundance of Ascomycota and the higher relative abundance of Basidiomycota in later years reflected year-associated shifts in the dominant fungal phyla. However, direct comparison of the magnitude of fungal and bacterial community reorganization would require formal cross-domain statistical testing.

### 3.7. Effects of Biochar Application on Soil Bacterial and Fungal Beta Diversity

**Figure 9:**
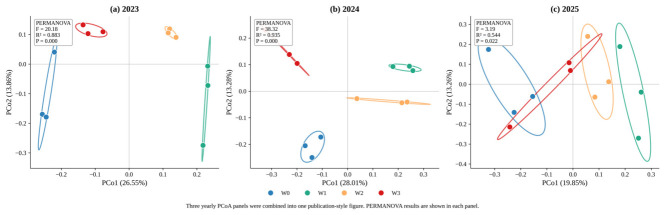
PCoA of bacterial community structure based on Bray–Curtis distance. Note: Each colored circle represents one replicate, and the ellipses indicate the 95% confidence intervals for each treatment group.

**Figure 10:**
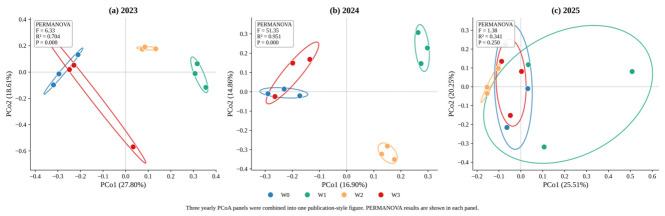
PCoA of fungal community structure based on Bray–Curtis distance. Note: Each colored circle represents one replicate, and the ellipses indicate the 95% confidence intervals for each treatment group.

Bray–Curtis-based principal coordinate analysis (PCoA) indicated year- and biochar application-rate-associated differences in bacterial and fungal community composition (Figure 9 and Figure 10). Bacterial communities exhibited partial clustering and separation among biochar application rates. In selected year-specific ordinations, W2 and W3 were separated from W0, consistent with treatment-associated differences in bacterial community composition. Sample distributions also differed among years, indicating that bacterial community composition varied across years, as well as among biochar application rates.

PERMANOVA of bacterial Bray–Curtis dissimilarities indicated significant effects of year, biochar application rate, and their interaction on bacterial community composition. Year explained the largest proportion of variation (F = 46.456, R^2^ = 0.667, *p* = 0.001). Biochar application rate also had a significant effect (F = 2.809, R^2^ = 0.060, *p* = 0.013), as did the year × biochar application rate interaction (F = 2.334, R^2^ = 0.101, *p* = 0.004). The significant interaction indicated that treatment-associated differences in bacterial community composition varied among years. In year-specific analyses, biochar application rate significantly affected bacterial community composition in 2023 (F = 2.984, R^2^ = 0.528, *p* = 0.001) and 2024 (F = 5.578, R^2^ = 0.677, *p* = 0.001), whereas the treatment effect was not significant in 2025 (F = 1.281, R^2^ = 0.324, *p* = 0.133).

Fungal PCoA likewise showed differences in sample distribution among years and biochar application rates. PERMANOVA indicated that year significantly affected fungal community composition (F = 31.626, R^2^ = 0.586, *p* = 0.001), and biochar application rate also had a significant effect (F = 2.116, R^2^ = 0.059, *p* = 0.020). The year × biochar application rate interaction was also significant (F = 2.381, R^2^ = 0.132, *p* = 0.004). In year-specific analyses, biochar application rate significantly affected fungal community composition in 2023 (F = 1.932, R^2^ = 0.420, *p* = 0.024) and 2024 (F = 3.680, R^2^ = 0.580, *p* = 0.001), whereas the treatment effect was not significant in 2025 (F = 1.332, R^2^ = 0.333, *p* = 0.165).

Overall, bacterial and fungal beta diversity was significantly associated with year, biochar application rate, and their interaction. In both datasets, year explained a substantially larger proportion of variation than biochar application rate (bacteria: R^2^ = 0.667 vs. 0.060; fungi: R^2^ = 0.586 vs. 0.059). Thus, interannual variation represented by year was the dominant source of compositional variation captured by the models, whereas biochar application rate explained a smaller but statistically significant component. Because year may reflect multiple unmeasured temporal factors, its effect should not be attributed to any single environmental driver.

### 3.8. Analysis of Differential Microbial Taxa Under Different Biochar Treatments

**Figure 11:**
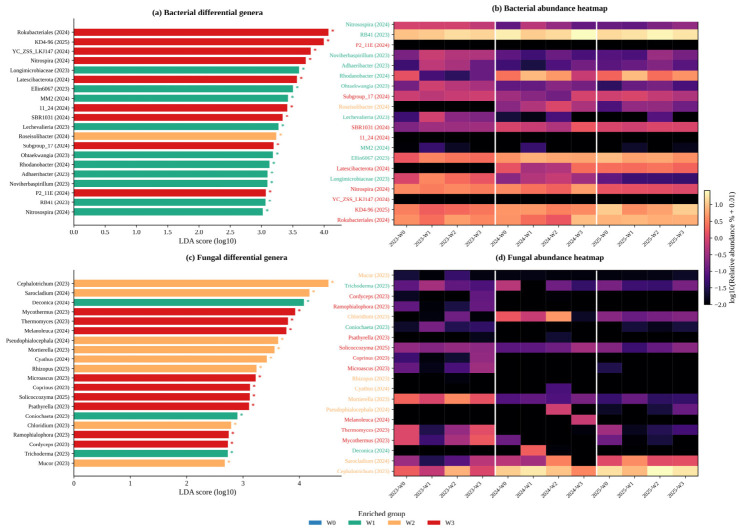
Heatmaps and LDA scores of key differential genera under biochar treatments. Note: * *p* < 0.05.

LEfSe analysis identified bacterial and fungal taxa that were differentially enriched among biochar application rates (Figure 11 and Supplementary Figures S1–S3), indicating treatment-associated differences in microbial community composition.

Within the bacterial community, W1 was associated with the enrichment of Longimicrobiaceae, Ellin6067, MM2, *Lechevalieria*, *Ohtaekwangia*, *Rhodanobacter*, *Adhaeribacter*, *Noviherbaspirillum*, RB41, and *Nitrosospira*. *Roseisolibacter* was differentially enriched under W2. W3 was associated with a larger set of discriminatory bacterial taxa, including Rokubacteriales, KD4-96, YC_ZSS_LKJ147, *Nitrospira*, Latescibacterota, SBR1031, Subgroup_17, and P2_11E. The high LDA scores of Rokubacteriales and KD4-96 under W3 indicate that these taxa made relatively large contributions to treatment discrimination in the LEfSe analysis; however, these scores should not be interpreted as direct measures of overall community dissimilarity.

Within the fungal community, W1 was associated with the enrichment of *Deconica*, *Coniochaeta*, and *Trichoderma*. W2 was associated with the enrichment of *Cephalotrichum*, *Sarocladium*, *Pseudophialocephala*, *Mortierella*, *Cyathus*, *Rhizopus*, *Chloridium*, and *Mucor*. W3 was associated with the enrichment of *Mycothermus*, *Thermomyces*, *Melanoleuca*, *Microascus*, *Coprinus*, *Solicoccozyma*, *Psathyrella*, *Ramophialophora*, and *Cordyceps*. Some taxa enriched under W2 have been reported in previous studies as potentially associated with saprotrophic activity or organic matter decomposition. However, their functional roles and in situ activities cannot be confirmed from ITS amplicon data alone.

Overall, the low, medium, and high biochar application rates were associated with distinct sets of discriminatory bacterial and fungal taxa. W3 showed a comparatively broader set of treatment-associated biomarkers in both bacterial and fungal communities. These findings support the conclusion that microbial community composition differed among biochar application rates. However, the specific environmental mechanisms underlying these differences cannot be determined solely from LEfSe results, and require direct measurements of soil environmental variables and microbial functional potential or activity.

### 3.9. Relationships Between Soil Environmental Factors and Microbial Community Structure

Before RDA, variance inflation factor (VIF) analysis was conducted to assess multicollinearity among the environmental variables. In the initial model, the VIF values for organic matter (OM), available potassium (AK), and pH were 55.20, 29.68, and 18.06, respectively. All values exceeded the commonly used threshold of 10, indicating substantial multicollinearity. After removal of OM, which had the highest VIF value, six environmental variables were retained in the final RDA model: AK, total phosphorus (TP), total potassium (TK), pH, available phosphorus (AP), and alkali-hydrolyzable nitrogen (AN). Their final VIF values were 5.69, 4.57, 3.41, 2.82, 2.77, and 2.55, respectively; all were below 10, indicating that multicollinearity was within an acceptable range in the final model.

**Figure 12:**
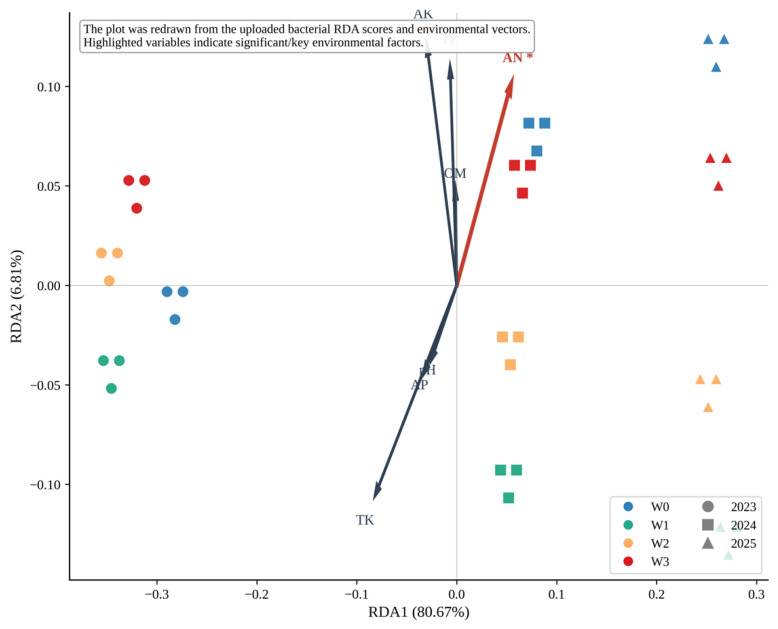
RDA of bacterial community and soil physicochemical factors. Note: * *p* < 0.05.

The overall bacterial RDA model was significant (F = 1.689, *p* = 0.043), and the retained environmental variables collectively explained 25.90% of the variation in bacterial community composition. This result (Figure 12) indicates that the measured soil nutrient variables and pH were jointly associated with bacterial community variation. In marginal permutation tests, AN was significant before false discovery rate (FDR) adjustment (F = 2.535, R^2^ = 0.065, *p* = 0.045). However, its Benjamini–Hochberg-adjusted *p* value was 0.264, and it was no longer significant after FDR correction. AK, pH, TK, TP, and AP were also not significant after FDR adjustment. Therefore, although the environmental variables collectively explained a significant proportion of bacterial community variation, no individual measured variable remained statistically significant after correction for multiple comparisons.

**Figure 13:**
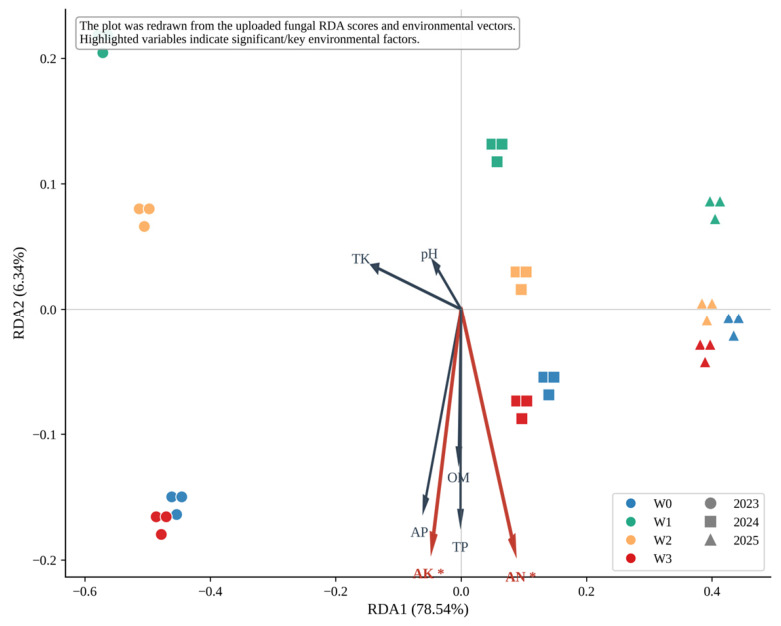
RDA of fungal community and soil physicochemical factors. Note: * *p* < 0.05.

The overall fungal RDA model (Figure 13) was not significant (F = 1.498, *p* = 0.079), although the retained environmental variables collectively explained 23.66% of the variation in fungal community composition. In marginal permutation tests, available potassium (AK) and alkali-hydrolyzable nitrogen (AN) explained relatively large proportions of variation (5.33% and 4.97%, respectively); however, their raw *p* values were 0.070 and 0.093, respectively, and neither association was statistically significant. After false discovery rate (FDR) adjustment, no individual environmental variable was significant. Accordingly, the measured soil physicochemical variables did not provide significant evidence for explaining fungal community variation in the present RDA. Potential contributions of unmeasured factors, including soil organic carbon fractions, rhizosphere conditions, interannual climatic variation, and time since biochar application, cannot be evaluated directly from the present dataset and should be examined in future studies.

**Figure 14:**
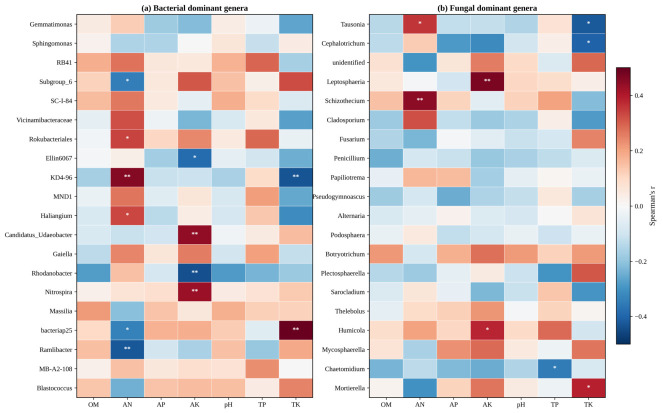
Correlations between dominant genera and soil physicochemical factors. Note: * *p* < 0.05; ** *p* < 0.01. Red indicates positive correlation.

Spearman correlation heatmaps showed statistical associations between dominant microbial genera and soil physicochemical variables (Figure 14). Among the dominant bacterial genera, KD4-96, *Candidatus Udaeobacter*, *Rhodanobacter*, and *Nitrospira* were significantly correlated with alkali-hydrolyzable nitrogen, available potassium, or total potassium. These results indicate that the relative abundances of several dominant bacterial taxa were associated with soil nitrogen- and potassium-related variables. Among the dominant fungal genera, *Leptosphaeria*, *Schizothecium*, *Tausonia*, *Cephalotrichum*, *Mortierella*, *Humicola*, and *Chaetomidium* were significantly correlated with alkali-hydrolyzable nitrogen, available potassium, total phosphorus, or total potassium. These results indicate that the relative abundances of several dominant fungal taxa were associated with measured soil nutrient variables. However, these correlations indicate statistical associations only, and do not establish causal relationships between microbial taxa and soil environmental factors.

Collectively, PERMANOVA, RDA, and correlation analyses indicated that bacterial and fungal community composition varied among biochar application rates and across years. PERMANOVA showed significant effects of year, biochar application rate, and their interaction, whereas year explained a substantially larger proportion of variation than biochar application rate. The bacterial RDA model was significant at the multivariable level, but no individual measured soil variable remained significant after false discovery rate (FDR) adjustment. In contrast, the fungal RDA model was not significant, and no individual measured environmental variable was significant after FDR adjustment.

Accordingly, the present analyses did not identify a single measured soil physicochemical variable that significantly explained microbial community variation after correction for multiple comparisons. Rather, they describe treatment- and year-associated microbial community patterns within the measured soil environmental context. Although correlation analyses identified statistical associations between selected dominant taxa and nutrient-related variables, these associations do not establish causal relationships or confirm the underlying ecological mechanisms. Direct measurements of soil carbon fractions, microhabitat properties, rhizosphere processes, and temporal climatic conditions would be required to clarify the drivers of microbial community variation following biochar application.

## 4. Discussion

### 4.1. Mechanisms by Which Biochar Improves Soil Fertility in Black Soil Maize Farmland

This study showed that biochar application was associated with substantial differences in soil physicochemical properties in maize farmland of the black soil region. Among the tested application rates, W3 was associated with the largest increases in measured soil organic matter, alkali-hydrolyzable nitrogen, available potassium, and pH, whereas W2 showed a comparatively balanced response across several nutrient indicators. The applied maize straw biochar contained 258.7 g kg^−1^ total carbon and had a cation-exchange capacity (CEC) of 38.1 cmolc kg^−1^, an alkaline pH of 8.89, an ash content of 18.60%, and a specific surface area of 86.35 m^2^ g^−1^. These batch-specific properties provide a physicochemical basis for considering potential contributions of the biochar to soil carbon inputs, nutrient retention, and pH regulation [13,14,15,16,17,18]. However, because the conventional soil organic matter assay used in this study cannot distinguish biochar-derived carbon from native soil organic matter, the increase in measured soil organic matter under W3 should be interpreted as an increase in the total measured soil organic matter pool following biochar application, rather than as direct evidence of newly formed native soil organic matter [21,43,44].

The effects of biochar on soil nutrient availability may be related to its nutrient content, pore structure, surface functional groups, and adsorption properties [14,15]. In the present study, the higher available potassium observed under W3 may partly reflect the potassium content of the maize-straw-derived biochar. The increases in alkali-hydrolyzable nitrogen under W2 and W3 were consistent with treatment-associated differences in soil nitrogen availability [25,45]. Although enhanced nutrient retention and altered microbial transformation processes could plausibly contribute to these patterns, neither process was directly measured in the present experiment. In addition to stable aromatic carbon, biochar may contain smaller labile-carbon fractions and surface-associated organic compounds that could influence dissolved organic carbon availability, microbial substrate accessibility, microbial biomass, and extracellular enzyme activity [15,18,19,20,21]. However, these active-carbon-related pathways were not directly quantified because dissolved organic carbon, microbial biomass carbon, enzyme activities, soil organic carbon fractions, and soil CO_2_ fluxes were not measured. They should therefore be regarded as plausible mechanisms rather than direct evidence of causal processes in the present field trial.

### 4.2. Responses of Bacterial Communities to Biochar Application and Their Ecological Significance

Bacterial alpha diversity patterns differed among biochar application rates and years, and did not exhibit a linear response to application rate [30]. In 2023, W3 showed comparatively high bacterial richness and diversity. This pattern was consistent with treatment-associated differences under the high biochar application rate during the first year of the experiment. Although biochar-induced changes in carbon inputs, soil pH, and nutrient availability could plausibly contribute to these differences [33,34], the present data do not directly identify the underlying mechanisms. In 2025, W2 showed higher Chao1, observed ASVs, and Shannon indices, indicating comparatively high bacterial richness and diversity under the medium application rate in the third year. This result does not establish that W2 is universally more suitable for maintaining bacterial diversity, but it suggests that bacterial alpha diversity responses differed between the medium and high biochar application rates over time.

At the phylum level, Proteobacteria, Acidobacteriota, Actinobacteriota, Gemmatimonadota, and Chloroflexi were among the dominant bacterial groups. At the genus or lower taxonomic levels, Gemmatimonas, RB41, KD4-96, and Nitrospira showed treatment-associated differences in relative abundance under the medium and high biochar application rates. These taxa may be associated with biochar-related changes in soil conditions, including carbon inputs, nutrient status, and pH [34,42,46]. However, their ecological functions cannot be directly inferred from taxonomic information or 16S rRNA gene amplicon data alone. Direct conclusions regarding nitrogen transformation, carbon metabolism, or other functional processes require verification using functional-gene analysis, metagenomic sequencing, or enzyme activity measurements.

Bacterial communities are often responsive to variation in soil pH and nutrient availability [31]. In the present study, biochar application was associated with higher soil pH and treatment-specific differences in several nutrient indicators, which may have contributed to the observed variation in bacterial community composition [17,19]. However, the present data do not directly demonstrate that the high biochar rate imposed stronger community filtering or that it selectively favored taxa adapted to higher pH or nutrient-rich conditions. From the perspective of the measured alpha diversity indices, W2 showed a comparatively balanced bacterial diversity response in 2025, whereas W3 was associated with relatively high diversity in 2023 and a distinct set of differentially enriched taxa. Further multi-year measurements of microbial function, soil carbon fractions, and crop performance are needed to determine the ecological and agronomic implications of these contrasting responses.

### 4.3. Responses of Fungal Communities to Biochar Application and Their Ecological Significance

Fungal alpha diversity patterns exhibited pronounced interannual variation and differed among biochar application rates [30,32]. In this study, fungal richness was comparatively high in 2023, declined in 2024, and partially recovered in 2025. In particular, W3 showed higher richness and diversity indices in 2025. These patterns indicate that fungal alpha diversity varied across years and biochar application rates. Although soil organic carbon fractions, pH, rhizosphere conditions, climatic variation, and time since biochar application may plausibly contribute to these patterns [32,37], their individual effects cannot be determined directly from the present dataset.

At the phylum level, Ascomycota remained the most abundant fungal phylum across all three years, although its relative abundance was lower in 2024 and 2025 than in 2023. In contrast, Basidiomycota showed a comparatively higher relative abundance in 2025. Previous studies have reported that many taxa within Ascomycota and Basidiomycota are associated with saprotrophic, plant-associated, or complex organic matter decomposition functions [47]. However, phylum-level relative abundance patterns cannot directly demonstrate changes in soil organic carbon decomposition processes or fungal niche distribution. At the genus level, Tausonia, Cephalotrichum, Leptosphaeria, Penicillium, and Mortierella differed among years and biochar application rates, indicating year- and treatment-associated variation in fungal community composition [36,39].

Fungal communities may contribute to soil carbon cycling and other farmland ecological processes, but their functional roles cannot be directly inferred from ITS amplicon data alone [48]. Some taxa identified in this study have been reported as potentially associated with saprotrophic activity or plant-associated functions [49]; however, their in situ activities and effects on crop health were not measured. In addition, phylum- or genus-level composition cannot reliably distinguish beneficial, neutral, and pathogenic fungi. Future studies should integrate fungal functional-guild annotation, targeted identification of plant-pathogenic fungi, soil enzyme activities, soil carbon fraction measurements, and crop health indicators to clarify the functional implications of biochar-associated shifts in fungal communities [50].

### 4.4. Differences Between Bacterial and Fungal Responses and Implications for Soil Health Assessment

This study showed clear differences in the responses of bacterial and fungal communities to biochar application. Bacterial alpha diversity was relatively well maintained under W2 and W3, whereas fungal alpha diversity exhibited stronger interannual fluctuations. PCoA results also showed that bacterial community shifts were relatively continuous, while fungal communities showed more distinct separation among years. These differences may be related to the contrasting ecological strategies of bacteria and fungi [7,48]. Bacteria generally have shorter growth cycles and respond rapidly to changes in available nutrients, pH, and moisture conditions, whereas fungi have well-developed hyphal structures and may be more sensitive to soil pore structure, organic matter composition, root exudates, and carbon-to-nitrogen balance [31,32].

From the perspective of farmland ecological functions, bacterial community changes may be more closely associated with nitrogen transformation, available nutrient supply, and certain carbon metabolic processes, whereas fungal community changes may contribute more to complex organic matter decomposition, soil aggregate formation, and rhizosphere interactions [7,48]. Therefore, evaluating the effects of biochar on the microecological environment of black soil requires consideration of both bacterial and fungal communities, rather than reliance on a single microbial group [30,33]. The present results suggest that biochar application was associated not only with changes in soil fertility indicators, but also with shifts in microbial community structure, thereby providing important implications for sustainable agricultural management in the black soil region [11,12].

### 4.5. Ecological Effects and Practical Feasibility of Different Biochar Application Rates

Different biochar application rates produced differentiated ecological effects [24,27]. W1 affected several soil indicators and microbial taxa, but showed limited overall improvement. W2 performed well in maintaining bacterial alpha diversity, improving several nutrient indicators, and enriching specific microbial taxa, suggesting favorable microecological regulation under the medium biochar rate [25,35]. W3 produced the strongest increases in measured soil organic matter, available potassium, alkali-hydrolyzable nitrogen, and pH, and was associated with more pronounced enrichment of differential bacterial and fungal taxa, suggesting a stronger effect of the high biochar rate on soil conditions and microbial community composition [22,26]. These results reveal an important ecological trade-off: W3 showed the greatest fertility-enhancement effect, whereas W2 appeared to provide a more balanced combination of soil improvement, microbial diversity maintenance, and practical feasibility.

However, the ecological effects of high-rate biochar application do not necessarily represent a linear enhancement with increasing application rate [27]. Although W3 markedly increased soil nutrient levels and pH, its effects on microbial diversity varied among years and may have imposed selective pressure on some microbial taxa by substantially altering soil pH and nutrient ratios. This may have contributed to the enrichment of certain dominant taxa and microbial community restructuring [35,36]. In addition, applying biochar at 40 t ha^−1^ may involve high costs and practical limitations in agricultural production [29]. Accordingly, within the soil fertility and microbial community indicators measured in the present experiment, W2 showed a comparatively balanced response relative to W3. This result should be regarded as a hypothesis for future yield– and cost–benefit trials, rather than as a recommendation for routine field application.

### 4.6. Mechanisms by Which Soil Environmental Factors Drive Microbial Community Shifts

PERMANOVA showed that year accounted for the largest proportion of variation in bacterial and fungal community structures, with explanatory rates of 66.68% and 58.63% for bacteria and fungi, respectively, which were clearly higher than the biochar treatment effect. This finding should not be interpreted as weakening the ecological relevance of biochar; rather, it indicates that microbial communities in open-field black soil systems are strongly shaped by annual climatic conditions, crop growth status, root inputs, and dynamic soil processes [8,9]. The significant treatment and year × treatment effects further suggest that biochar exerted a sustained modulatory influence, the intensity and direction of which depended on the annual environmental background [24,37].

RDA further showed that, after VIF screening, the retained environmental variables, including available potassium, total phosphorus, total potassium, pH, available phosphorus, and alkali-hydrolyzable nitrogen, explained 25.90% of bacterial community variation, and the overall bacterial model was significant. However, no single environmental variable remained significant after FDR correction, indicating that bacterial community shifts were more likely associated with the combined influence of soil nutrient status and pH than with any isolated variable. The overall fungal RDA model was not significant, suggesting that fungal communities may be associated with additional drivers not fully represented by the measured physicochemical variables, such as organic carbon fractions, root exudates, soil moisture, crop developmental status, and interannual climatic variation [32,49].

Regarding the potential pathway of biochar-associated microbial restructuring, biochar may first modify soil organic matter and nutrient availability through exogenous carbon and mineral nutrient inputs [13,21]. The batch-specific total carbon content, CEC, ash content, and alkaline pH of the applied biochar provide material properties that may contribute to these changes. Biochar may also regulate soil pH through alkaline ash components and influence water and nutrient retention through its pore structure and ion-exchange properties [15,17]. These changes can create new habitat conditions and resource gradients that influence microbial selection and community assembly [31,42]. However, the direct role of active carbon cannot be resolved from the current dataset because DOC, microbial biomass carbon, extracellular enzyme activity, SOC fractions, and gas fluxes were not measured. The proposed pathway should therefore be interpreted as a mechanistic framework consistent with the observed associations, not as a directly verified causal sequence.

RDA and correlation analyses primarily reveal statistical associations between environmental variables and community structure, and cannot fully demonstrate direct causality. Future studies should integrate soil enzyme activities, microbial biomass, functional genes, metagenomic sequencing, and crop yield data to construct a more complete mechanistic chain linking biochar input, soil environmental change, microbial functional response, and crop productivity [41,51].

### 4.7. Implications of Biochar Application for Sustainable Soil Ecosystem Management

Black soil conservation and sustainable maize production are important priorities for agricultural development in Northeast China [1,2]. This study suggests that biochar application can improve soil fertility in black soil maize farmland and is associated with shifts in bacterial and fungal community structures, indicating its potential as a soil amendment for fertility enhancement and microecological regulation in the black soil region [22,24]. From the perspective of sustainable management, rational biochar application has three main implications. First, biochar may increase measured soil organic matter and enhance nutrient retention capacity, thereby helping to alleviate fertility degradation in black soils [4,21]. Second, biochar may contribute to the regulation of bacterial and fungal community structures and support soil microecological processes [30,33]. Third, the use of biochar derived from agricultural residues provides a potential pathway for residue recycling, carbon sequestration, and resource utilization [13,21].

Nevertheless, no biochar rate should be interpreted as a universal practical recommendation on the basis of the present data alone. Although W3 markedly improved several soil nutrient indicators, it also represented a boundary-response scenario with a high straw-mass equivalent, and may impose greater material and application costs as well as stronger selective effects on microbial communities [24,29]. Within the soil fertility and microbial community indicators evaluated in this study, W2 showed a comparatively balanced response relative to W3. However, crop yield, economic feasibility, residue-supply logistics, carbon-fraction dynamics, and long-term ecological outcomes were not quantified; therefore, neither W2 nor W3 can be defined here as the agronomic optimum or recommended as a routine management rate. Future multi-site trials should integrate yield, cost–benefit, and carbon-process measurements before practical rate recommendations are made.

### 4.8. Limitations and Future Research

Several limitations should be acknowledged. First, the increase in measured soil organic matter could not be separated into biochar-derived carbon and newly accumulated native soil organic matter. Future studies should include carbon fractionation, stable-isotope tracing, or microbial biomass carbon analysis to clarify carbon transformation pathways. Second, although the applied biochar batch was characterized for total carbon and CEC, this study did not directly measure DOC, microbial biomass carbon, extracellular enzyme activity, SOC fractions, or soil CO_2_ fluxes; active-carbon-mediated mechanisms therefore remain to be tested. Third, microbial functions were inferred mainly from taxonomic composition; functional prediction, enzyme assays, functional gene quantification, or metagenomic sequencing are needed to verify the ecological roles of key bacterial and fungal taxa. Fourth, crop yield, economic feasibility, and residue-supply logistics were not evaluated, and the high-rate treatment had a large straw-mass equivalent. Hence, the present data cannot establish an agronomic or economic optimum. Finally, the strong year effect observed in PERMANOVA indicates that longer-term and multi-site monitoring is needed to distinguish persistent biochar effects from interannual climatic variation.

## 5. Conclusions

(1) Biochar application significantly affected soil physicochemical properties in maize fields of the black soil region. W3 produced the largest increases in measured soil organic matter, alkali-hydrolyzable nitrogen, available potassium, and pH, whereas W2 provided more consistent improvements across multiple fertility indicators.
(2) Bacterial and fungal alpha diversity responded differently to biochar application. Bacterial diversity was better maintained under W2 and W3, whereas fungal diversity exhibited stronger interannual variation and increased under W3 in 2025.
(3) Biochar application was associated with shifts in microbial community composition. The bacterial community was dominated by Proteobacteria, Acidobacteriota, Actinobacteriota, Gemmatimonadota, and Chloroflexi, whereas the fungal community was dominated by Ascomycota, Basidiomycota, and Mortierellomycota.
(4) Soil nutrient status and pH were associated with microbial community differentiation; however, no single measured factor remained significant after false discovery rate correction. These results suggest that biochar-associated microbial community shifts were more closely related to combined changes in soil conditions than to any single independent environmental driver.
(5) Within the soil fertility and microbial community indicators measured in this three-year field experiment, W2 showed a comparatively balanced response relative to W3. However, crop yield, economic feasibility, residue-supply logistics, labile carbon fractions, microbial biomass, enzyme activities, and gas fluxes were not quantified. Therefore, this study cannot define an agronomic or economic optimum biochar rate, nor can it directly verify active-carbon-mediated mechanisms.

## Figures and Tables

**Table 1:** Basic properties of the biochar used in the experiment.

Parameter	Value
Feedstock	Maize straw
Pyrolysis condition (°C)	500
Dry-mass biochar yield (%)	30.2
Particle size	<2 mm
Organic matter (g·kg^−1^)	447.07
Total C (g·kg^−1^)	258.7
Total N (g·kg^−1^)	6.01
Total P (g·kg^−1^)	5.89
Total K (g·kg^−1^)	27.61
pH	8.89
Ash content (%)	18.60
C/N	43.10
CEC cmol(+) kg^−1^	38.1
Specific surface area (m^2^·g^−1^)	86.35

Note: All values are expressed on a dry-weight basis. Biochar yield was calculated as dry mass of biochar recovered divided by dry mass of input maize straw × 100.

**Table 2:** Linear mixed model analysis of the effects of year, biochar treatment, and their interaction on soil physicochemical properties.

Soil Property	Fixed Effect	Chi-Squared	df	*p* Value	Significance
Organic matter	Year	5.648	2	0.059	ns
Organic matter	Treatment	2266.489	3	<0.001	***
Organic matter	Year × Treatment	21.331	6	0.002	**
Alkali-hydrolyzable N	Year	293.901	2	<0.001	***
Alkali-hydrolyzable N	Treatment	222.995	3	<0.001	***
Alkali-hydrolyzable N	Year × Treatment	1989.526	6	<0.001	***
Available P	Year	152.964	2	<0.001	***
Available P	Treatment	131.087	3	<0.001	***
Available P	Year × Treatment	237.549	6	<0.001	***
Available K	Year	1.423	2	0.491	ns
Available K	Treatment	256.499	3	<0.001	***
Available K	Year × Treatment	44.148	6	<0.001	***
Total P	Year	7.591	2	0.022	*
Total P	Treatment	181.119	3	<0.001	***
Total P	Year × Treatment	75.441	6	<0.001	***
pH	Year	15.284	2	<0.001	***
pH	Treatment	663.097	3	<0.001	***
pH	Year × Treatment	35.968	6	<0.001	***
Total N	Year	0.41	2	0.815	ns
Total N	Treatment	669.606	3	<0.001	***
Total N	Year × Treatment	448.641	6	<0.001	***
Total K	Year	17.124	2	<0.001	***
Total K	Treatment	1.281	3	0.734	ns
Total K	Year × Treatment	17.162	6	0.009	**

Note: Linear mixed models were used to test the effects of year, biochar treatment, and their interaction. * *p* < 0.05; ** *p* < 0.01; *** *p* < 0.001; ns, not significant.

**Table 3:** PERMANOVA analysis of the effects of year, biochar treatment, and their interaction on bacterial and fungal community structures.

Microbial Group	Dataset	Factor	F Value	R^2^	*p* Value	Significance
Bacteria	Overall	Year	46.456	0.667	0.001	**
Bacteria	Overall	Treatment	2.809	0.06	0.013	*
Bacteria	Overall	Year × Treatment	2.334	0.101	0.004	**
Bacteria	2023	Treatment	2.984	0.528	0.001	**
Bacteria	2024	Treatment	5.578	0.677	0.001	**
Bacteria	2025	Treatment	1.281	0.324	0.133	ns
Fungi	Overall	Year	31.626	0.586	0.001	**
Fungi	Overall	Treatment	2.116	0.059	0.02	*
Fungi	Overall	Year × Treatment	2.381	0.132	0.004	**
Fungi	2023	Treatment	1.932	0.42	0.024	*
Fungi	2024	Treatment	3.68	0.58	0.001	**
Fungi	2025	Treatment	1.332	0.333	0.165	ns

Note: PERMANOVA was performed based on Bray–Curtis distance with 999 permutations. * *p* < 0.05; ** *p* < 0.01; ns, not significant.

**Table 4:** Sequencing quality of bacterial and fungal communities under different biochar treatments across years.

Microbial Group (Year)	Treatment	Replicates	Effective Sequences	ASV Number	Good’s Coverage
Bacteria (2023)	W0	3	94,160 ± 18,764	4654.4 ± 361.6	0.9907 ± 0.0031
Bacteria (2023)	W1	3	90,976 ± 28,546	4786.4 ± 896.5	0.9902 ± 0.0062
Bacteria (2023)	W2	3	102,775 ± 2808	5065.5 ± 78.4	0.9876 ± 0.0003
Bacteria (2023)	W3	3	115,063 ± 7324	5773.9 ± 516.4	0.9835 ± 0.0032
Fungi (2023)	W0	3	101,186 ± 28,500	807.6 ± 168.9	0.9992 ± 0.0006
Fungi (2023)	W1	3	105,559 ± 12,513	557.3 ± 69.8	0.9993 ± 0.0003
Fungi (2023)	W2	3	117,149 ± 10,286	805.2 ± 53.3	0.9989 ± 0.0000
Fungi (2023)	W3	3	90,291 ± 20,486	712.0 ± 245.6	0.9994 ± 0.0006
Bacteria (2024)	W0	3	40,394 ± 2405	3319.1 ± 106.2	0.9945 ± 0.0018
Bacteria (2024)	W1	3	43,738 ± 1376	3016.3 ± 78.4	0.9936 ± 0.0007
Bacteria (2024)	W2	3	41,740 ± 1160	3055.3 ± 172.7	0.9945 ± 0.0009
Bacteria (2024)	W3	3	39,211 ± 1082	3276.5 ± 204.3	0.9955 ± 0.0007
Fungi (2024)	W0	3	64,175 ± 5908	204.3 ± 7.7	0.9999 ± 0.0001
Fungi (2024)	W1	3	68,724 ± 4432	158.9 ± 6.6	0.9998 ± 0.0000
Fungi (2024)	W2	3	72,332 ± 23,250	191.9 ± 6.1	0.9998 ± 0.0001
Fungi (2024)	W3	3	54,190 ± 7323	173.1 ± 19.7	0.9999 ± 0.0001
Bacteria (2025)	W0	3	34,134 ± 0	2917.3 ± 129.2	0.9906 ± 0.0015
Bacteria (2025)	W1	3	34,134 ± 0	3069.0 ± 222.7	0.9884 ± 0.0019
Bacteria (2025)	W2	3	34,134 ± 0	3209.7 ± 268.2	0.9881 ± 0.0038
Bacteria (2025)	W3	3	34,134 ± 0	3118.0 ± 248.7	0.9881 ± 0.0017
Fungi (2025)	W0	3	54,063 ± 0	263.3 ± 28.0	0.9999 ± 0.0001
Fungi (2025)	W1	3	54,063 ± 0	240.0 ± 38.3	0.9999 ± 0.0001
Fungi (2025)	W2	3	54,063 ± 0	247.7 ± 54.0	0.9999 ± 0.0000
Fungi (2025)	W3	3	54,063 ± 0	282.3 ± 43.3	0.9999 ± 0.0001

Note: Values are presented as mean ± standard deviation. The sampling year is indicated in parentheses after each microbial group. ASV, amplicon sequence variant.

## Data Availability

The original data presented in the study are openly available in NCBI Sequence Read Archive (SRA) at PRJNA1471445, SRP704337, SAMN60444155-SAMN60444190 and SRR38875725-SRR38875796.

## Supplementary Materials

The following supporting information can be downloaded at: https://www.mdpi.com/article/10.3390/microorganisms14071487/s1, Figure S1: LEfSe analysis of bacterial and fungal communities under different biochar application rates in 2023; Figure S2: LEfSe analysis of bacterial and fungal communities under different biochar application rates in 2024; Figure S3: LEfSe analysis of bacterial and fungal communities under different biochar application rates in 2025.
