# Supplementary figures and images for: Rate-Dependent Effects of Biochar on Soil Fertility and Bacterial–Fungal Communities in Maize Fields of the Black Soil Region: A Three-Year Field Study
Source: Microorganisms. 2026 Jul 7;14(7):1487. doi: 10.3390/microorganisms14071487 (PMC13414337; doi:10.3390/microorganisms14071487)

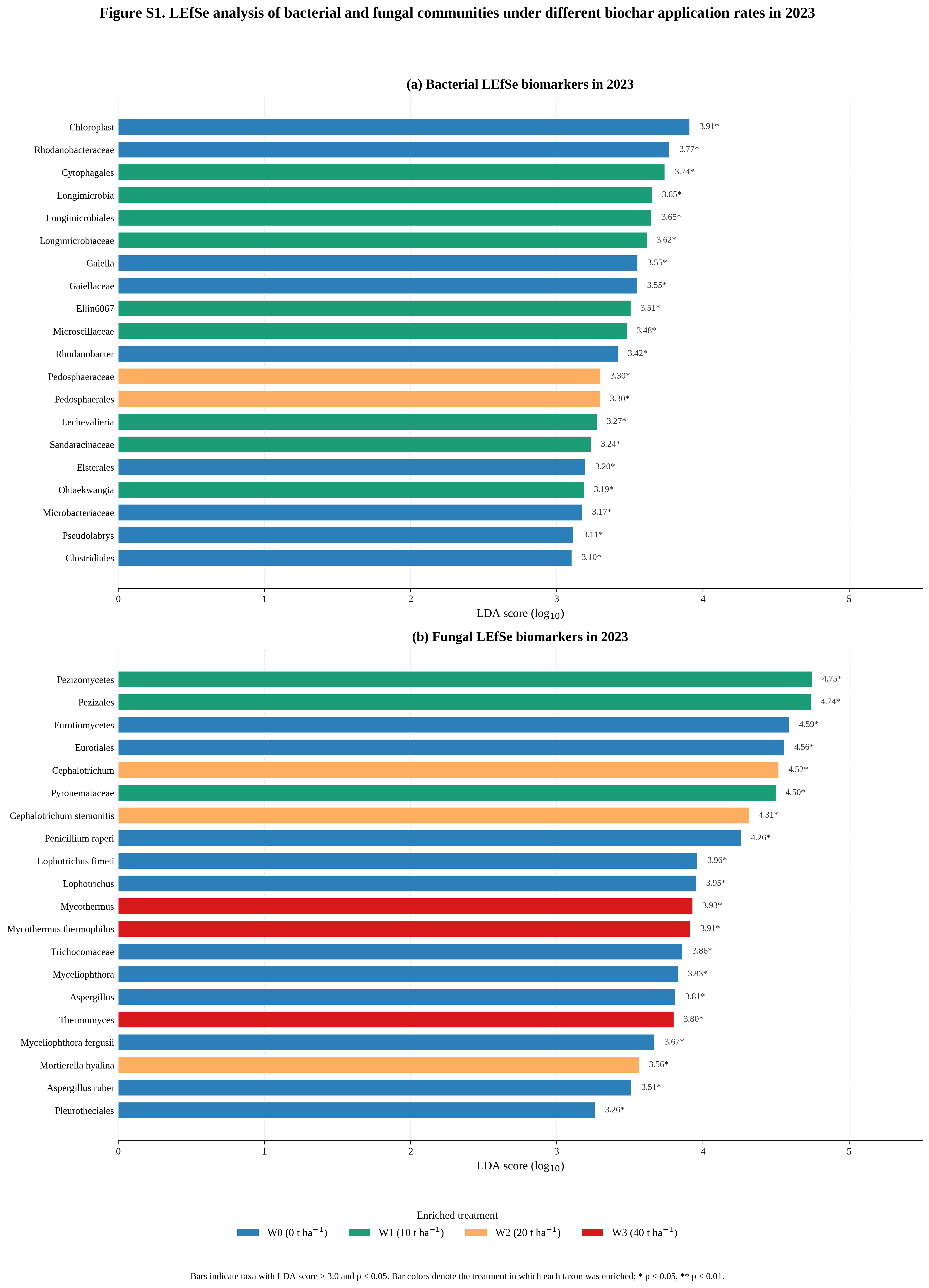

Supplement: Supplementary file 1 [file microorganisms-14-01487-s001.zip › Figure_S1_2023_LEfSe_300dpi.png]

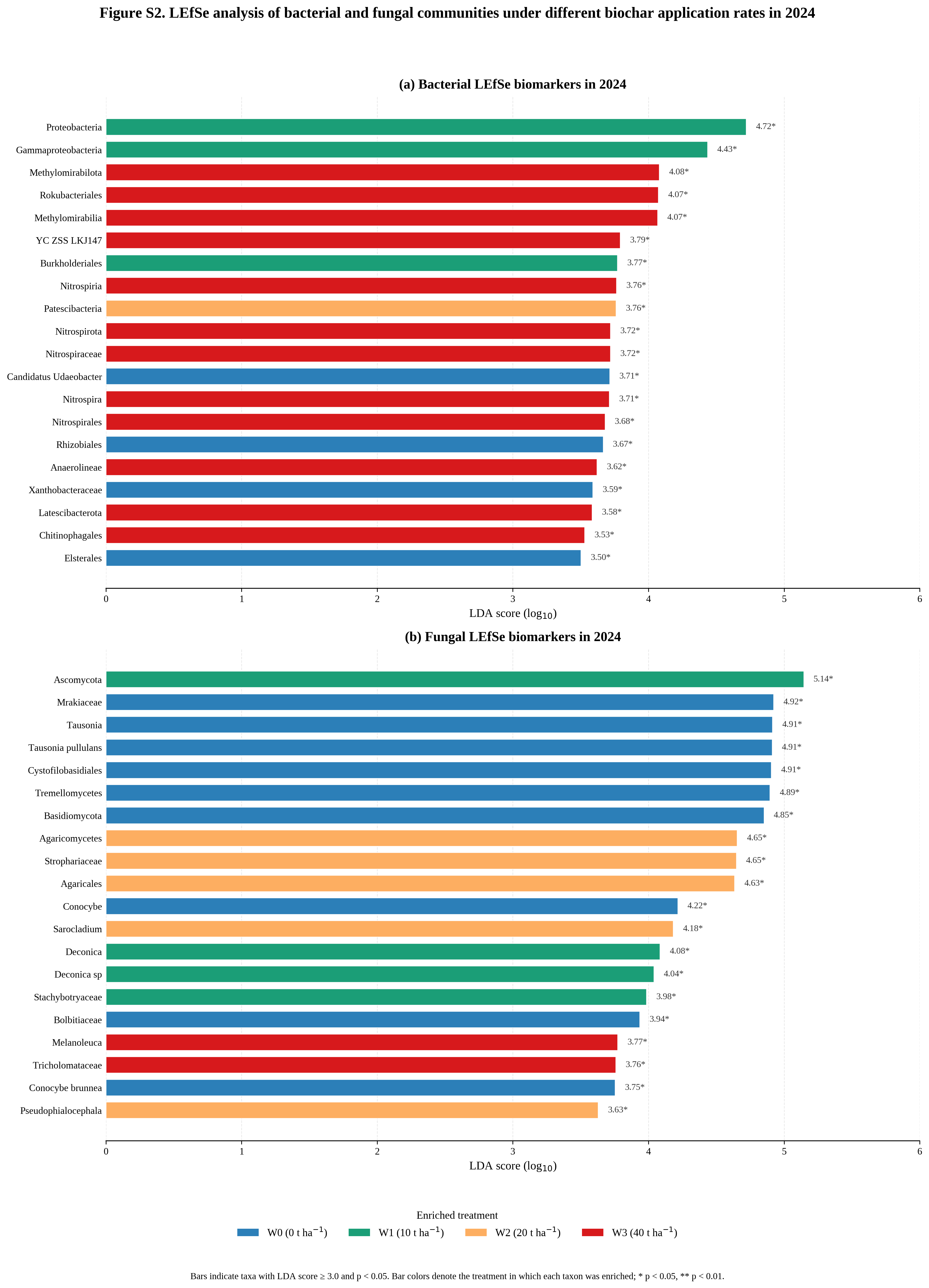

Supplement: Supplementary file 1 [file microorganisms-14-01487-s001.zip › Figure_S2_2024_LEfSe_300dpi.png]

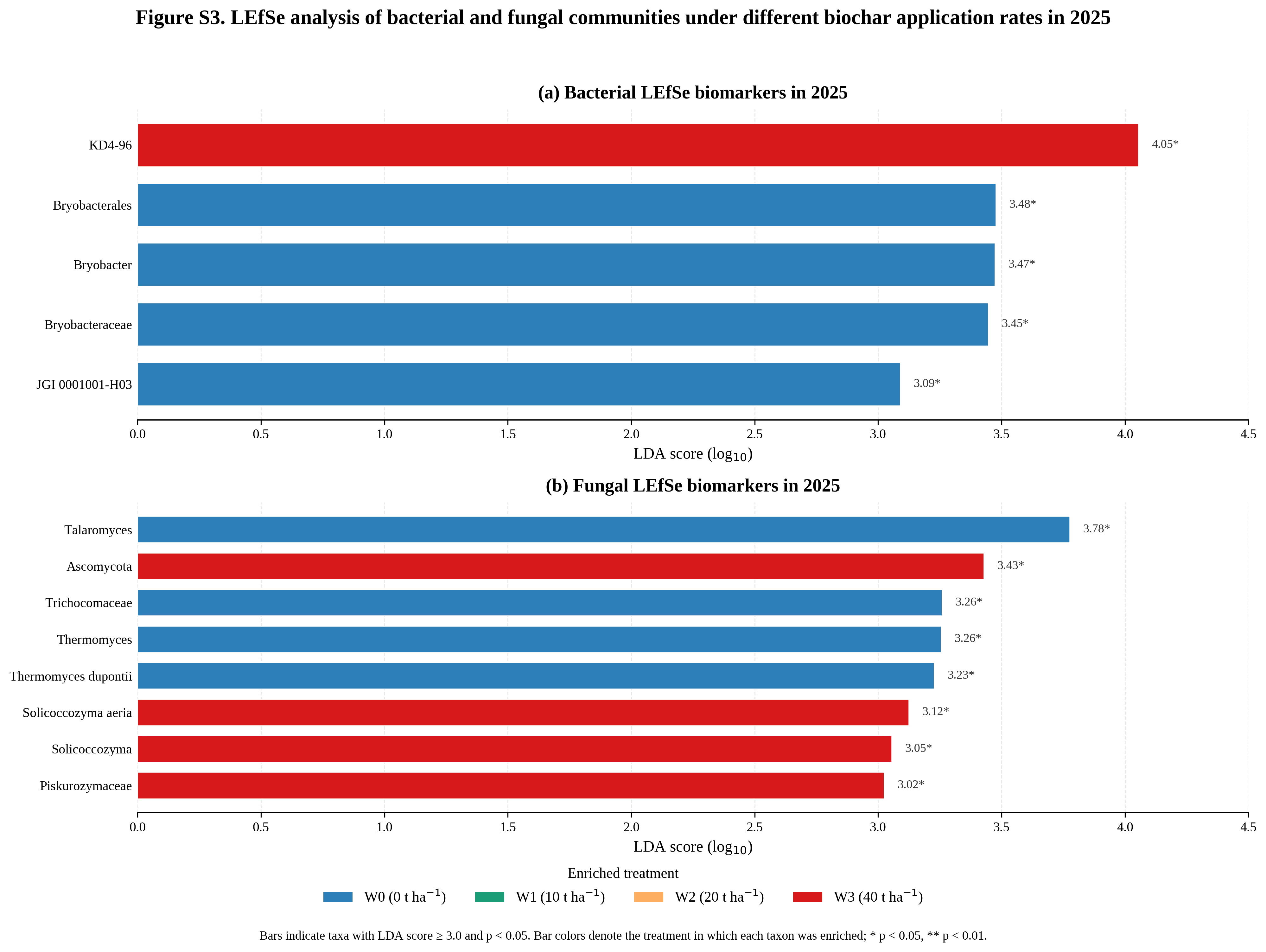

Supplement: Supplementary file 1 [file microorganisms-14-01487-s001.zip › Figure_S3_2025_LEfSe_300dpi.png]
